# Two new species of *Scytinostroma* (Russulales, Basidiomycota) in Southwest China

**DOI:** 10.3389/fcimb.2023.1189600

**Published:** 2023-05-18

**Authors:** Qiu-Yue Zhang, Hong-Gao Liu, Lu-Sen Bian, Qian Chen

**Affiliations:** ^1^ Institute of Microbiology, School of Ecology and Nature Conservation, Beijing Forestry University, Beijing, China; ^2^ Yunnan Key Laboratory of Gastrodia and Fungi Symbiotic Biology, Zhaotong University, Zhaotong, Yunnan, China; ^3^ Experimental Centre of Forestry in North China, Warm Temperate Zone Forestry Jiulong Mountain National Permanent Scientific Research Base, Chinese Academy of Forestry, Beijing, China; ^4^ College of Architecture and Urban Planning, Chongqing Jiaotong University, Chongqing, China; ^5^ College of Architecture and Urban Planning, Tongji University, Shanghai, China

**Keywords:** new taxa, Peniophoraceae, phylogeny, taxonomy, wood-rotting fungi

## Abstract

Two new species of *Scytinostroma* viz. *S. acystidiatum* and *S. macrospermum*, are described from southwest China. Phylogeny based on ITS + nLSU dataset demonstrates that samples of the two species form two independent lineages and are different in morphology from the existing species of *Scytinostroma*. *Scytinostroma acystidiatum* is characterized by resupinate, coriaceous basidiomata with cream to pale yellow hymenophore, a dimitic hyphal structure with generative hyphae bearing simple septa, the absence of cystidia, and amyloid, broadly ellipsoid basidiospores measuring 4.7–7 × 3.5–4.7 μm. *Scytinostroma macrospermum* is characterized by resupinate, coriaceous basidiomata with cream to straw yellow hymenophore, a dimitic hyphal structure with generative hyphae bearing simple septa, numerous cystidia embedded or projecting from hymenium, and inamyloid, ellipsoid basidiospores measuring 9–11 × 4.5–5.5 μm. The differences between the new species and morphologically similar and phylogenetically related species are discussed.

## 1 Introduction

The genus *Scytinostroma* Donk (Russulales, Basidiomycota), typified by *S. portentosum* (Berk. & M.A. Curtis) Donk, was established by Donk (1956). It is traditionally characterized by resupinate, coriaceous basidiomata, smooth to tuberculate hymenophore and a dimitic hyphal structure with simple septa or clamps on generative hyphae, filiform and dichotomously branched skeletal hyphae which are dextrinoid and cyanophilous, and subglobose to ellipsoid, thin-walled, variably amyloid basidiospores, and a white-rotting ecology (Donk, 1956; Rattan, 1974; Bernicchia and Gorjón, 2010; Wang et al., 2020; Tabish and Daniel, 2021).

The genus accommodated seven species derived from *Corticium* Fr. (without gloeocystidia) and *Gloeocystidium* P. Karst. (with gloeocystidia) when it was established. Later, *Scytinostroma* was gradually recognized by taxonomists, and the number of new species and new combinations has been increasing continuously (Donk, 1956; Gilbertson, 1962; Boidin, 1967; Rattan, 1974; Boidin and Lanquetin, 1977; Lanquetin, 1984; Boidin and Lanquetin, 1987; Boidin and Gilles, 1988; Hjortstam, 1990; Stalpers, 1996). So far, 36 species have been accepted in *Scytinostroma* worldwide (Nakasone, 2008; Liu, 2019; Wang et al., 2020). Recently, molecular phylogenetic studies demonstrated that *Scytinostroma* nested in Peniophoraceae within Russulales; furthermore, *Scytinostroma* was polyphyletic and formed four stable clades, as well as related to *Gloiothele* Bres., *Vararia* P. Karst., and *Dichostereum* Pilát (Nakasone and Micales, 1988; Larsson and Larsson, 2003; Miller et al., 2006; Larsson, 2007). Morphologically, *Scytinostroma* species are separated from other corticioid fungi of Russulales mainly by their tough and leathery texture of the basidiomata, as well as dextrinoid and dichotomously branched skeletal hyphae (Rattan, 1974; Liu, 2019).

During investigations on the diversity of wood-rotting fungi from China, two unknown corticioid specimens were collected from southwest China, and their morphology corresponded to the concepts of *Scytinostroma*. To confirm their affinity, phylogenetic analyses based on the ITS+ nLSU rDNA sequences were carried out. The two newly sequenced samples from Guizhou and Chongqing formed two well-supported lineages clustered with two sequences from Korea (KJ668461, Jang et al., 2016) and Japan (LC327052, Ogura-Tsujita et al., 2018), respectively. Based on morphological and phylogenetic evidences, we hereby propose two new species of *Scytinostroma*.

## 2 Materials and methods

### 2.1 Morphological studies

The studied specimens are deposited in the herbarium of the Institute of Microbiology, Beijing Forestry University (BJFC). Macro-morphological descriptions are based on field notes and dried specimens. Color terms followed Petersen (1996). Microscopic structures and abbreviations used in this study followed Wu et al. (2020) and Liu et al. (2022).

### 2.2 DNA extraction and sequencing

A CTAB rapid plant genome extraction kit (Aidlab Biotechnologies, Co., Ltd., Beijing, China) was used to obtain DNA products from voucher specimens, according to the manufacturer’s instructions with some modifications (Yuan et al., 2021; Yuan et al., 2022). The following primer pairs were used to amplify the DNA: ITS5 (5′-GGA AGT AAA AGT CGT AAC AAG G-3′) and ITS4 (5′-TCC TCC GCT TAT TGATAT GC-3′) for the internal transcribed spacer (ITS) regions (White et al., 1990); LR0R (5′-ACC CGC TGA ACT 6 TAA GC-3′) and LR7 (5′-TAC TAC CAC CAA GAT CT-3′) for nuclear large subunit (nLSU) rDNA (Vilgalys and Hester, 1990).

The procedures for DNA extraction and polymerase chain reaction (PCR) used in this study were the same as described by Wu et al. (2022b). The PCR products were purified and sequenced by Beijing Genomics Institute (BGI), China. All newly generated sequences in this study were deposited in GenBank (http://www.ncbi.nlm.nih.gov/genbank/) and listed in **Table 1**.

**Table 1:** Taxa information and GenBank accession numbers of sequences used in this study.

Species	Specimen no.	Locality	ITS	nLSU	Literature
*Confertobasidium olivaceoalbum*	FP 90196	USA	AF511648	AF511648	Larsson and Larsson, 2003
*Metulodontia nivea*	NH 13108	Russia	AF506423	AF506423	Larsson and Larsson, 2003
***Scytinostroma acystidiatum***	**Dai 24608**	**China**	**OQ689127**	**OQ629351**	**Present study**
*S. acystidiatum*	KUC20121019-32	Korea	KJ668461	−	Jang et al., 2016
*S. aluta*	CBS 762.81	France	MH861482	MH873221	Vu et al., 2019
*S. alutum*	CBS 763.81	France	MH861483	MH873222	Vu et al., 2019
*S. alutum*	CBS 764.81	France	MH861484	MH873223	Vu et al., 2019
*S. alutum*	CBS 765.81	France	MH861485	MH873224	Vu et al., 2019
*S. alutum*	CBS 766.81	France	MH861486	MH873225	Vu et al., 2019
*S. caudisporum*	CBS 746.86	Gabon	MH862030	NG073580	Vu et al., 2019
*S. crispulum*	CBS 716.86	Reunion	MH862013	MH873703	Vu et al., 2019
*S. crispulum*	CBS 717.86	France	MH862014	MH873704	Vu et al., 2019
*S. crispulum*	CBS 718.86	France	MH862015	MH873705	Vu et al., 2019
*S. crispulum*	CBS 776.86	France	MH862053	MH873741	Vu et al., 2019
*S. decidens*	CBS 714.86	France	MH862011	MH873701	Vu et al., 2019
*S. decidens*	CBS 715.86	France	MH862012	MH873702	Vu et al., 2019
*S. duriusculum*	CBS 757.81	France	MH861477	MH873216	Vu et al., 2019
*S. duriusculum*	CBS 758.81	France	MH861478	MH873217	Vu et al., 2019
*S. hemidichophyticum*	CBS 702.84	Belgium	MH861818	MH873509	Vu et al., 2019
*S. hemidichophyticum*	CBS 759.81	France	MH861479	MH873218	Vu et al., 2019
*S. hemidichophyticum*	CBS 760.81	France	MH861480	MH873219	Vu et al., 2019
*S. jacksonii*	NH 6626	Canada	AF506467	AF506467	Larsson and Larsson, 2003
*S. jacksonii*	CBS 239.87	Canada	MH862071	MH873759	Vu et al., 2019
***S. macrospermum***	**Dai 24606**	**China**	**OQ689126**	**OQ629350**	**Present study**
*S. macrospermum*	M2138	Japan	LC327052	−	Ogura‐Tsujita et al., 2018
*S. mediterraneense*	CBS 764.86	France	MH862045	MH873732	Vu et al., 2019
*S. mediterraneense*	CBS 765.86	France	MH862046	MH873733	Vu et al., 2019
*S. mediterraneense*	CBS 766.86	France	MH862047	MH873734	Vu et al., 2019
*S. microspermum*	CBS 238.87	Guadeloupe	MH862070	−	Vu et al., 2019
*S. ochroleucum*	CBS 767.86	France	MH862048	−	Vu et al., 2019
*S. ochroleucum*	CBS 768.86	France	MH862049	MH873735	Vu et al., 2019
*S. ochroleucum*	CBS 126049	USA	MH864062	MH875517	Vu et al., 2019
*S. odoratum*	KHL 8546	Sweden	AF506469	AF506469	Larsson and Larsson, 2003
*S. phaeosarcum*	CBS 728.81	Cote d’Ivoire	MH861481	MH873205	Vu et al., 2019
*S. portentosum*	CBS 503.48	Canada	MH856447	MH873220	Vu et al., 2019
*S. pseudopraestans*	CBS 737.91	−	MH862322	MH873994	Vu et al., 2019
*S. pseudopraestans*	CBS 738.91	−	MH862323	MH873995	Vu et al., 2019
*S. pseudopraestans*	CBS 739.91	−	MH862324	MH873996	Vu et al., 2019
*S. pseudopraestans*	CBS 740.91	−	MH862325	MH873997	Vu et al., 2019
*S. pseudopraestans*	CBS 741.91	−	MH862326	MH873998	Vu et al., 2019
*S. pseudopraestans*	CBS 742.91	−	MH862327	−	Vu et al., 2019
*S. quintasianum*	CBS 749.86	Cote d’Ivoire	MH862031	MH873719	Vu et al., 2019
*S. quintasianum*	CBS 750.86	−	MH862032	MH873720	Vu et al., 2019
*S. quintasianum*	CBS 751.86	−	MH862033	−	Vu et al., 2019
*S. renisporum*	CBS 771.86	Indonesia	MH862051	MH873738	Vu et al., 2019
*S. renisporum*	CBS 772.86	Indonesia	MH862052	MH873739	Vu et al., 2019
*S. yunnanense*	CLZhao 10758	China	MT611445	−	Wang et al., 2020
*S. yunnanense*	CLZhao 10802	China	MT611446	−	Wang et al., 2020
*S. yunnanense*	CLZhao 11010	China	MT611447	−	Wang et al., 2020
*S.* sp1	KUC20130725-13	Korea	KJ668460	−	Jang et al., 2016
*S.* sp2	MEL:2382679	Australia	KP013042	−	Rosenthal et al., 2017
*S.* sp3	UC2022985	USA	KP814265	−	Rosenthal et al., 2017
*S.* sp3	UC2022946	USA	KP814564	−	Rosenthal et al., 2017
*S.* sp4	MEL:2382745	Australia	KP012928	−	Rosenthal et al., 2017
*S.* sp5	LR-40	Chile	MT366713	−	Direct Submission
*S.* sp6	Het 803-1	USA	OL989828	−	Otto et al., 2021
*S.* sp6	NO 6-1-B	USA	OK173822	−	Otto et al., 2021
*S.* sp6	iNAT:30809947	USA	MZ267776	−	Direct Submission
*S.* sp7	UoA SVB-F86	−	MT975590	−	Direct Submission
*S.* sp7	UC2023098	Canada	KP814402	−	Rosenthal et al., 2017

The new species are in bold. "-" represents the absence of a certainsequence in the species.

### 2.3 Phylogenetic analyses

Phylogenetic analyses were performed with the Maximum Parsimony (MP), Maximum Likelihood (ML), and Bayesian Inference (BI) methods. New sequences generated in this study, along with reference sequences retrieved from GenBank (**Table 1**), were aligned by MAFFT 7 (Katoh et al., 2019; http://mafft.cbrc.jp/alignment/server/) using the “G-INS-i” strategy and manually adjusted in BioEdit (Hall, 1999). Unreliably aligned sections were removed before the analyses, and efforts were made to manually inspect and improve the alignment. The data matrix was edited in Mesquite v3.70. *Confertobasidium olivaceoalbum* (Bourdot & Galzin) Jülich and *Metulodontia nivea* (P. Karst.) Parmasto were selected as outgroups (Larsson and Larsson, 2003).

MP topology and bootstrap (BT) values obtained from 1,000 replicates were computed in PAUP* version 4.0b10 (Swofford, 2002). All characters were equally weighted, and the gaps were treated as missing data. Trees were inferred using the heuristic search option with tree-bisection reconnection (TBR) branch swapping and 1,000 random sequence additions. Max-trees were set to 5,000, branches of zero length were collapsed, and all parsimonious trees were saved. Clade robustness was assessed by a BT analysis with 1,000 replicates (Felsenstein, 1985). Descriptive tree statistics, such as tree length (TL), consistency index (CI), retention index (RI), rescaled consistency index (RC), and homoplasy index (HI) were calculated for each Maximum Parsimonious Tree (MPT) generated.

RAxML 7.2.8 was used to construct ML trees for the combined dataset with the GTR+I+G model of site substitution, including estimation of Gamma-distributed rate heterogeneity and a proportion of invariant sites (Stamatakis, 2006). The branch support was evaluated with a bootstrapping method of 1000 replicates (Hillis and Bull, 1993).

The BI was conducted with MrBayes 3.2.6 in two independent runs, each of which had four chains for 5 million generations and started from random trees (Ronquist and Huelsenbeck, 2003). Trees were sampled every 1,000 generations. The first 25% of the sampled trees were discarded as burn-in, and the remaining ones were used to reconstruct a majority rule consensus and calculate Bayesian Posterior Probabilities (BPP) of the clades.

Branches that received BT supports for Maximum Parsimony (BP) and Maximum Likelihood (BS) greater than or equal to 75%, and BPP greater than or equal to 0.95 were considered as significantly supported. FigTree v1.4.4 and Treeview (Page, 1996) were used to visualize the resulting tree.

## 3 Results

### 3.1 Phylogenetic results

**Figure 1:**
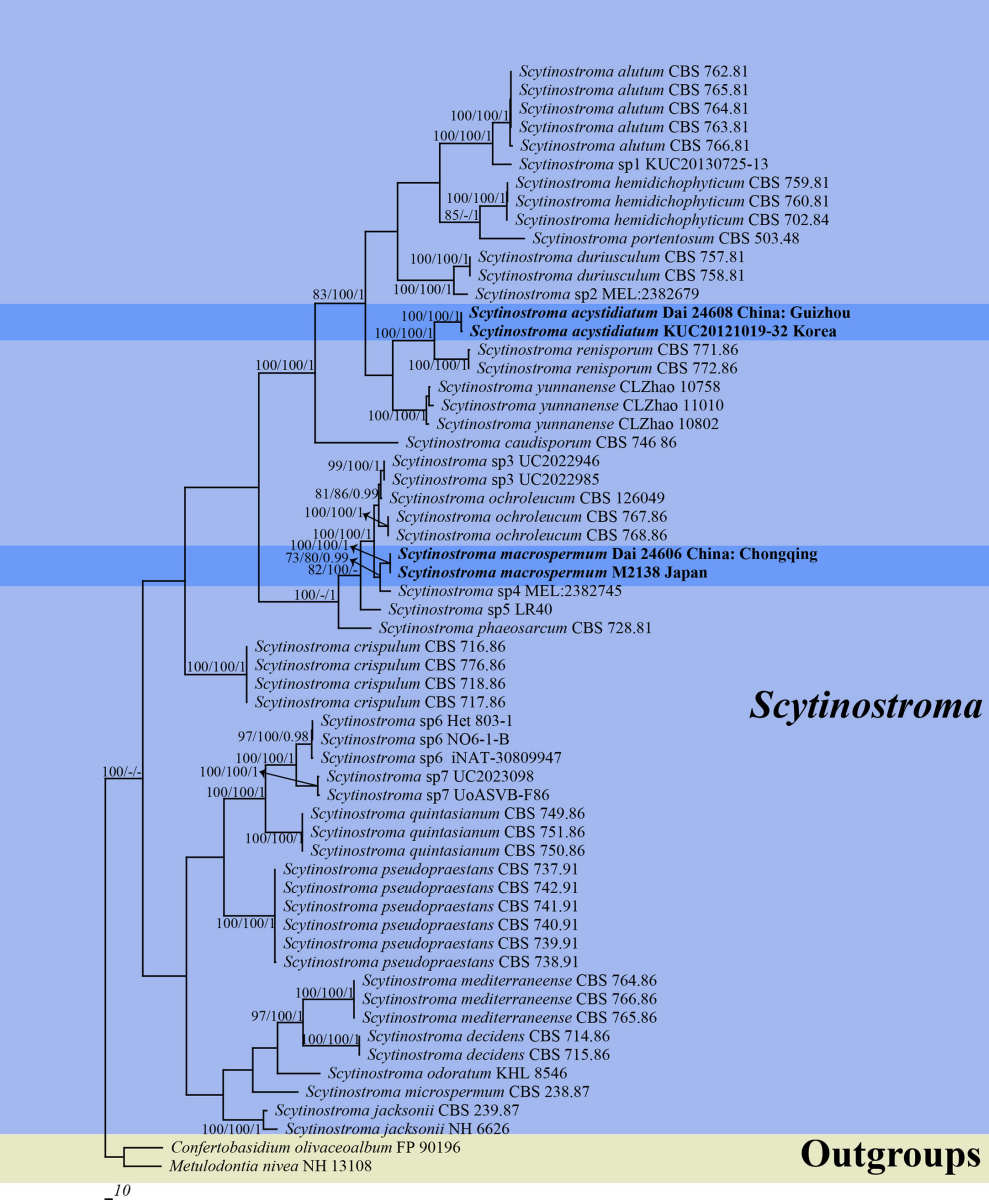
Phylogeny of *Scytinostroma* by Maximum Parsimony (MP) analysis based on combined ITS + nLSU dataset. Branches are labeled with bootstrap supports for Maximum Parsimony (BP) > 70%, Maximum Likelihood bootstrap (BS) > 70%, and Bayesian Posterior Probabilities (BPP) > 0.95, respectively. The new species are in bold.

Two ITS and two nLSU sequences were generated in this study and were deposited in GenBank. Their accession numbers are specified in the phylogenetic tree (**Figure 1**). The final ITS + nLSU dataset included 60 sequences representing 28 species and resulted in an alignment of 1,826 characters. Maximum parsimony analysis yielded one equally parsimonious tree (TL = 2833, CI = 0.502, HI = 0.846, RI = 0.424, and RC = 0.498). BI analysis and ML analysis resulted in a similar topology to the MP analysis, with an average standard deviation of split frequencies of 0.002601 (BI).

The phylogeny (**Figure 1**) inferred from the ITS + nLSU dataset demonstrated that two new species, *Scytinostroma acystidiatum* and *S. macrospermum*, clustered in the *Scytinostroma* clade. Moreover, *Scytinostroma acystidiatum* clustered with one sample from Korea (KUC20121019-32) formed an independent lineage with a robust support (BP = 100%, BS = 100%, and BPP = 1.00) and then closely related to *S. renisporum* Boidin, Lanq. & Gilles. *S. macrospermum* clustered with one sample from Japan (M2138), forming an independent lineage with a strong support (BP = 100%, BS = 100%, and BPP = 1.00).

### 3.2 Taxonomy

**Figure 2:**
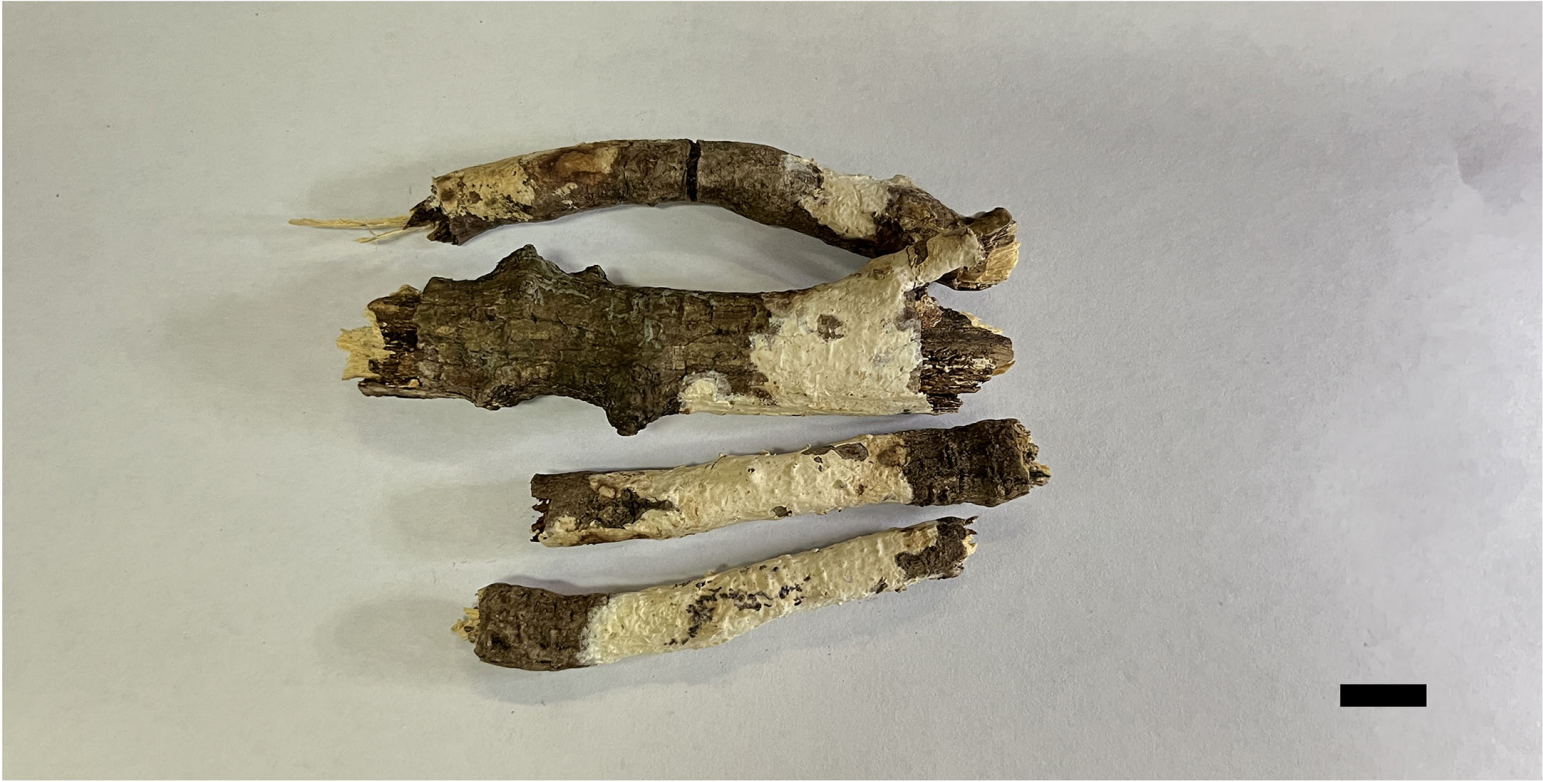
Basidiomata of *Scytinostroma acystidiatum* (Holotype, Dai 24608). Scale bar = 1.0 cm. Photo by: Qiu-Yue Zhang.

**Figure 3:**
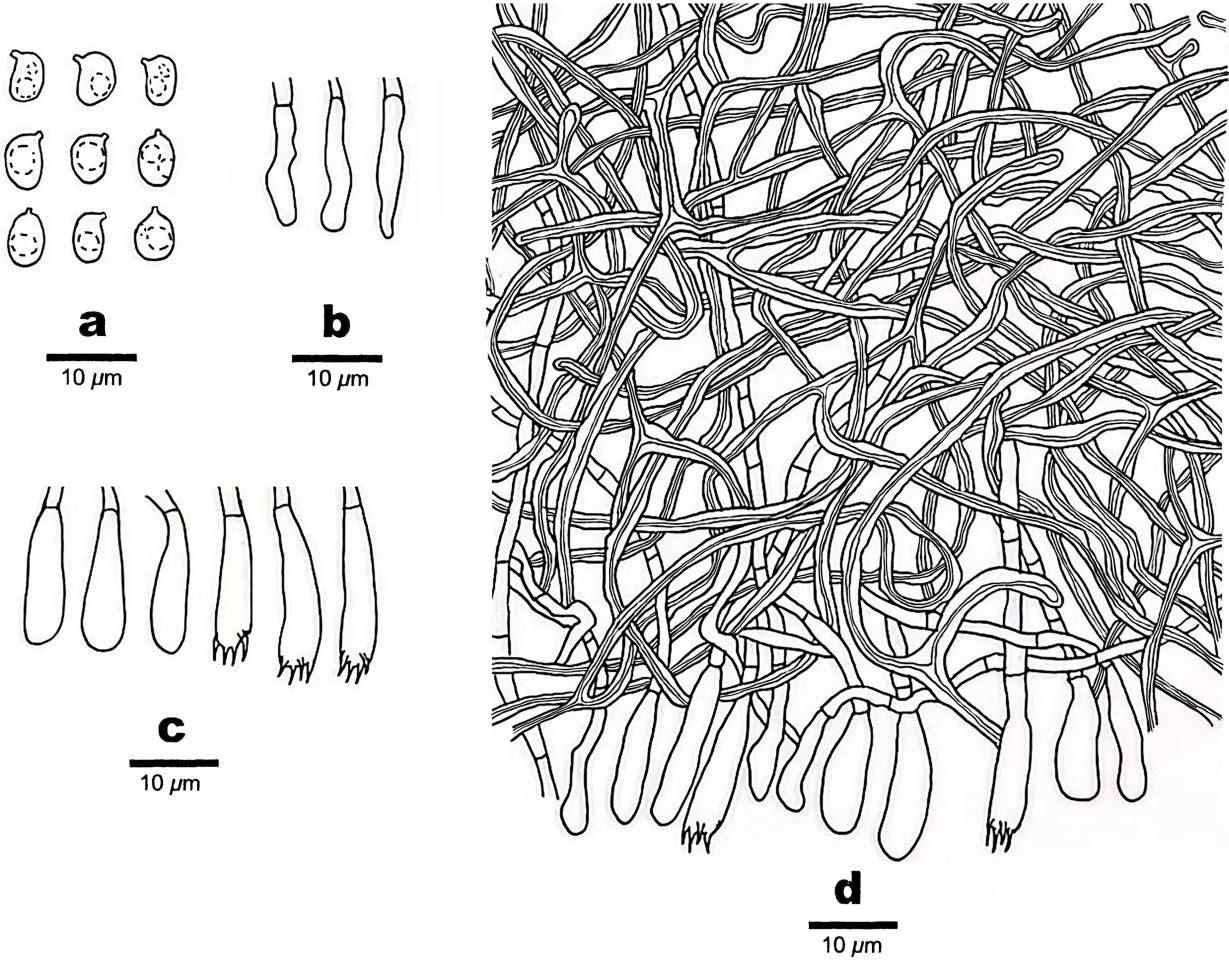
Microscopic structures of *Scytinostroma acystidiatum* (Holotype, Dai 24608). **(A)** Basidiospores. **(B)** Cystidioles. **(C)** Basidia and basidioles. **(D)** A section of basidiomata. Drawings by: Qiu-Yue Zhang.

*Scytinostroma acystidiatum* Q.Y. Zhang, L.S. Bian & Q. Chen, sp. nov., **Figures 2**, **3**

MycoBank no.: 848524

*Type* — China, Guizhou Province, Tongren, Fanjingshan, on fallen angiosperm branch, 13 July 2022, Dai 24608 (BJFC039842).

*Etymology* **—** *Acystidiatum* (Lat.): refers to the species lacking cystidia.

*Basidiomata* —Annual, resupinate, coriaceous, not separable from substrate, up to 7 cm long, 2 cm wide, and less than 0.1 mm thick at center. Hymenial surface smooth to locally tuberculate, cream to pale yellow; margin concolorous with hymenial surface, thinning out, and adnate.

*Hyphal structure* —Hyphal system dimitic; generative hyphae infrequent, simple septate, hyaline, thin-walled, rarely branched, 2–3 μm in diameter, IKI–, CB–; skeletal hyphae dominant, frequently dichotomously branched, tortuous, interwoven, thick-walled, dextrinoid, cyanophilous, 1–2.5 μm in diameter; tissues unchanged in KOH.

*Hymenium* —Cystidia absent; cystidioles present, clavate, some gradually tapering to the apex, thin-walled, hyaline, smooth, 12–18 × 2–4 μm; basidia clavate, with a basal simple septum and four sterigmata, thin-walled, smooth, 13–21 × 3.5–5 μm; basidioles similar to basidia in shape, but slightly smaller.

*Spores* —Basidiospores broadly ellipsoid with an apiculus, hyaline, thin-walled, smooth, occasionally with one or two guttules, amyloid, acyanophilous, (4.5–)4.7–7 × (3–)3.5–4.7(–5) μm, *L* = 5.68 μm, *W* = 4.02 μm, *Q* = 1.41 (*n* = 30/1).

**Figure 4:**
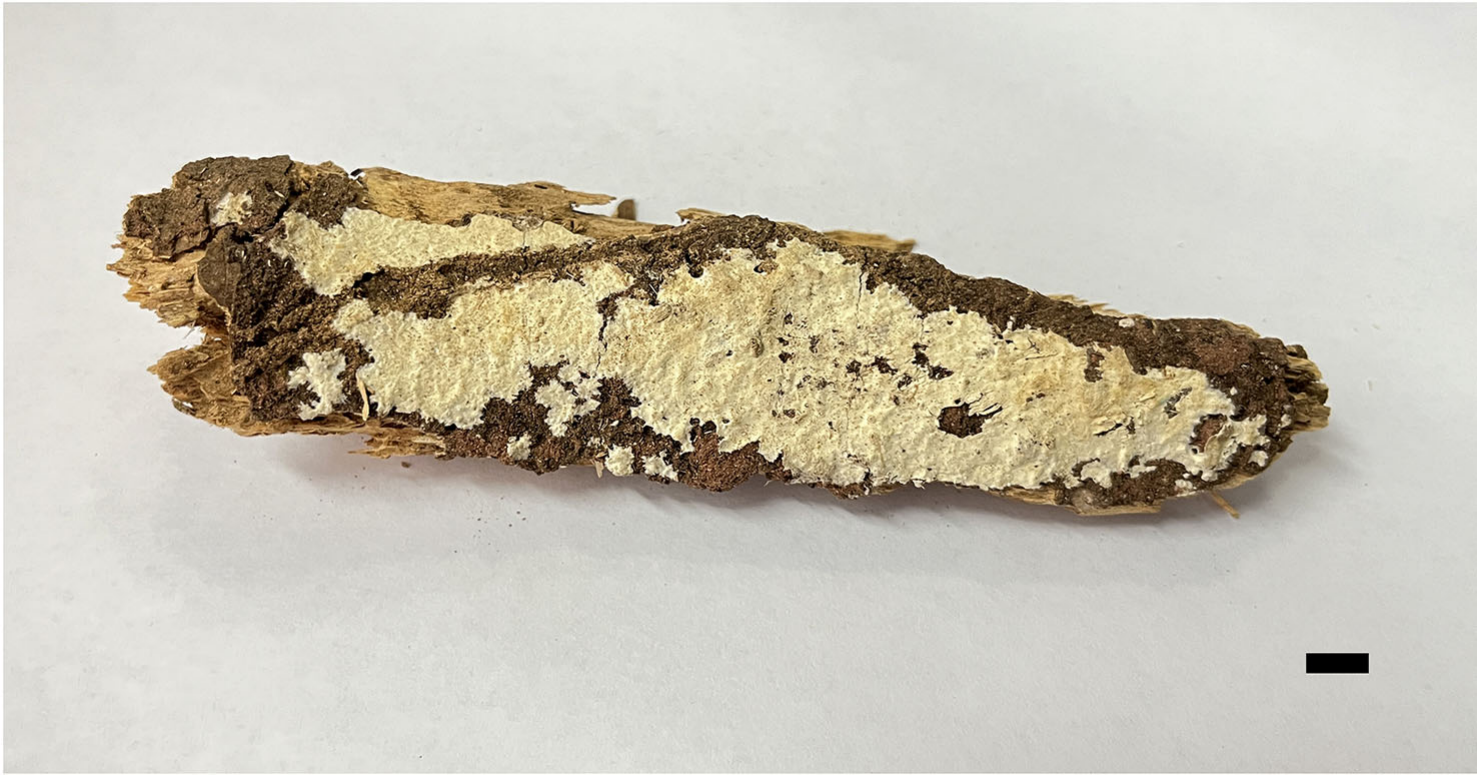
Basidiomata of *Scytinostroma macrospermum* (Holotype, Dai 24606). Scale bar = 1.0 cm. Photo by: Qiu-Yue Zhang.

**Figure 5:**
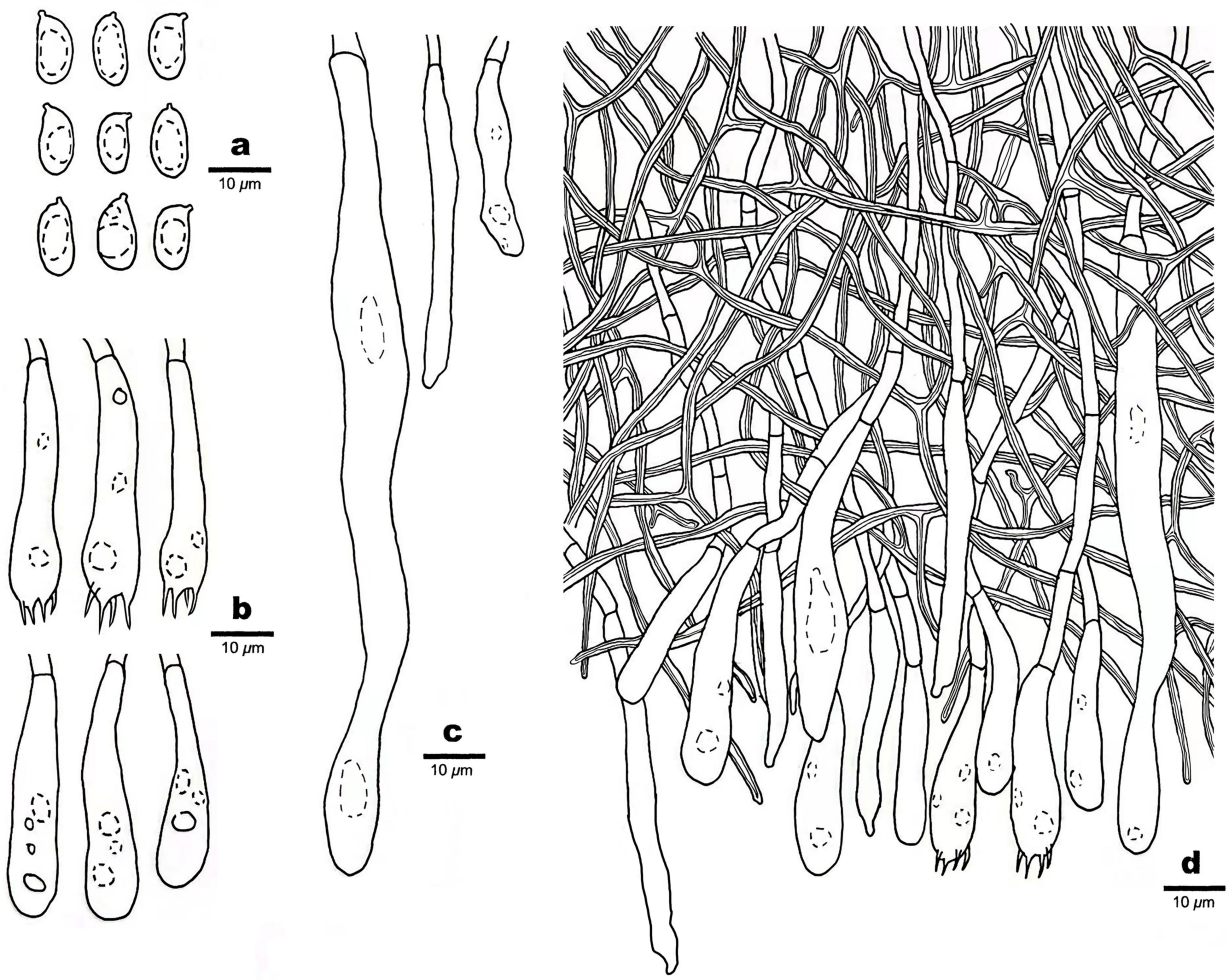
Microscopic structures of *Scytinostroma macrospermum* (Holotype, Dai 24606). **(A)** Basidiospores. **(B)** Basidia and basidioles. **(C)** Cystidia. **(D)** A section of basidiomata. Drawings by: Qiu-Yue Zhang.

*Scytinostroma macrospermum* Q.Y. Zhang, L.S. Bian & Q. Chen, sp. nov., **Figures 4**, **5**

MycoBank no.: 848525

*Type* —China, Chongqing, Jiangjin District, Simianshan National Scenic Spot, on rotten angiosperm wood, 10 July 2022, Dai 24606 (BJFC039838).

*Etymology* — *Macrospermum* (Lat.): refers to the species having large basidiospores.

*Basidiomata* —Annual, resupinate, coriaceous, not separable from substrate, up to 13.5 cm long, 3 cm wide, and less than 0.2 mm thick at center. Hymenial surface smooth to locally tuberculate, cream to straw yellow; margin concolorous with hymenial surface, slightly fimbricate.

*Hyphal structure* —Hyphal system dimitic; generative hyphae infrequent, simple septate, thin-walled, hyaline, rarely branched, 1.5–3 μm in diameter, IKI–, CB–; skeletal hyphae dominant, frequently dichotomously branched, interwoven, thick-walled, dextrinoid, cyanophilous, 1–3 μm in diameter; tissues unchanged in KOH.

*Hymenium* —Cystidia numerous, narrowly fusoid to cylindrical, thin-walled, smooth, 25–107 × 2.5–10 μm, embedded or projecting from hymenium up to 25 µm; basidia clavate, with a basal simple septum and four sterigmata, thin-walled, smooth, with some guttules, 30–45 × 6–8 μm; basidioles dominant, similar to basidia in shape, but slightly smaller.

*Spores* —Basidiospores ellipsoid with an apiculus, hyaline, thin-walled, smooth, occasionally with one or two guttules, inamyloid, acyanophilous, 9–11(–12) × (4–)4.5–5.5(–6) μm, *L* = 9.89 μm, *W* = 4.94 μm, *Q* = 2.00 (*n* = 30/1).

## 4 Discussion

Two new species, *Scytinostroma acystidiatum* and *S. macrospermum*, are described in this study based on morphological characteristics and phylogenetic analyses. The ITS + nLSU-based phylogeny (**Figure 1**) shows the phylogenetic positions of the two new species in the genus *Scytinostroma*. In detail, the sequence of KUC20121019-32 from Korea, clustered together with *Scytinostroma acystidiatum*, and shares less than 1.5% sequence (ITS) dissimilarity (Jang et al., 2016). The sample KUC20121019-32 was collected in Odaesan National Park, South Korea, which has geographical proximity (eastern Asia) and a similar climate (subtropical climate) to Guizhou, China. So, we treat KUC20121019-32 as *Scytinostroma acystidiatum*. In addition, *Scytinostroma acystidiatum* grouped with *S. renisporum* with strong support (100% BP, 100% BS, 1.00 BPP, **Figure 1**). *Scytinostroma renisporum* is morphologically distinguished from *S. acystidiatum* by its membranaceous to paper-like basidiomata and larger gloeocystidia measuring 20–35 × 6–10 µm (Boidin and Lanquetin, 1987).

Morphologically, *Scytinostroma alutum* Lanq., *S. arachnoideum* (Peck) Gilb., *S. cystidiatum* Boidin, *S. hemidichophyticum* Pouzar, *S. portentosum* (Berk. & M.A. Curtis) Donk, and *S. yunnanense* C.L. Zhao are similar to *S. acystidiatum* by sharing amyloid basidiospores. However, *S. alutum* differs from *S. acystidiatum* by its resupinate to effuse-reflexed basidiomata with cracked hymenophore, larger basidia (40–65 × 5–6 µm *vs.* 13–21 × 3.5–5 μm), and bigger basidiospores (5.3–7.2 × 5.7–7.3 μm *vs.* 4.7–7 × 3.5–4.7 μm; Lanquetin, 1984). *Scytinostroma arachnoideum* is separated from *S. acystidiatum* by its cottony basidiomata with white rhizomorphs and smaller basidiospores (3.5–4.5 × 3–3.5 μm *vs.* 4.7–7 × 3.5–4.7 μm; Gilbertson, 1962). *Scytinostroma cystidiatum*, *S. hemidichophyticum*, and *S. portentosum* are separated from *S. acystidiatum* by the presence of cystidia (Donk, 1956; Boidin, 1960; Pouzar, 1966). *S. yunnanense* differs from *S. acystidiatum* by its white to cream basidiomata and shorter basidiospores (4.5–5.5 μm *vs.* 4.7–7 μm in length; Wang et al., 2020).

Phylogenetically, the sequence of M2138 from Japan, clustered together with *Scytinostroma macrospermum* and formed an independent lineage with less than 1.5% sequence (ITS) dissimilarity (Ogura-Tsujita et al., 2018). The sample M2138 was collected in Kagoshima, Japan, which has geographical proximity (eastern Asia) and a similar climate (subtropical climate) to Chongqing, China. So, we treat M2138 as *Scytinostroma macrospermum* (**Figure 1**). Morphologically, *Scytinostroma ochroleucum* (Bres. & Torrend) Donk resembles *S. macrospermum* by resupinate, cream-colored to pale ochraceous basidiomata, but the former is different from the latter by its larger basidia (35–85 × 6.5–9 µm *vs.* 30–45 × 6–8 μm), and larger basidiospores (9–14 × 5–7 µm *vs.* 9–11 × 4.5–5.5 μm; Donk, 1956). *Scytinostroma phaeosarcum* Boidin & Lanq. resembles *S. macrospermum* by the approximately same size of basidiospores (8–10 × 4.5–5.5 μm), while *S. phaeosarcum* differs from *S. macrospermum* by its basidiomata becoming brown when bruised and thinner basidia (3–5 μm *vs.* 6–8 μm in width; Boidin and Lanquetin, 1977). In addition, *Scytinostroma macrospermum* is similar to S*. decidens* Boidin, Gilles & Lanq., *S. jacksonii* Boidin and *S. mediterraneense* Boidin & Lanq. by sharing large cystidia (> 100 μm in length) and inamyloid basidiospores. However, the latter three species distinctly differ from *S. macrospermum* by their obviously narrower basidiospores (2.5–3.5 μm in width *vs.* 4.5–5.5 μm in width, Boidin, 1981; Boidin and Lanquetin, 1987; Nakasone and Micales, 1988).

Wood-rotting fungi as an important group within the Basidiomycota are known for their ecological role in the forest ecosystem in terms of decaying living and dead trees and recycling nutrients in forest ecosystems (Dai et al., 2007; Yuan et al., 2021; Yuan et al., 2022). However, the diversity and taxonomy of these fungi remain not well known, and many new species have been described recently because of the application of molecular phylogeny (Dai et al., 2021; Mao et al., 2023; Wang et al., 2021; Wang et al., 2022; Wu et al., 2022a; Wu et al., 2022b; Zhou et al., 2021). Similarly, despite numerous species of *Scytinostroma* have been described, many unknown species or unnamed sequences still exist (*Scytinostroma* sp., **Figure 1**). Consequently, with the application of molecular phylogeny, the diversity and systematics will be outlined by further studies based on more samples worldwide.

## Data availability statement

The datasets presented in this study can be found in online repositories. The names of the repository/repositories and accession number(s) can be found below: https://www.ncbi.nlm.nih.gov/genbank/, OQ629350, OQ629351, OQ689126, OQ689127.

## Author contributions

Q-YZ, H-GL, L-SB, and QC designed the research and contributed to data analysis and interpretation. Q-YZ prepared the samples and drafted the manuscript. H-GL, L-SB and QC discussed the results and edited the manuscript. All authors contributed to the article and approved the submitted version.
